# Adding salt to foods, genetic susceptibility, and risk of incident osteoporosis

**DOI:** 10.1016/j.jnha.2026.100973

**Published:** 2026-09-05

**Authors:** Xukun Luo, Jiayin Li, Hongxuan Chen, Juan Duan, Tang Liu, Jian Zhou

**Affiliations:** a Department of Orthopedics, The Second Xiangya Hospital of Central South University, Changsha, China; b Clinical Medicine Eight-year Program, 2302 Class, 2023 Grade, Central South University, Changsha, China; c National Clinical Research Center for Metabolic Diseases, The Second Xiangya Hospital of Central South University, Changsha, China; d Clinical Medicine Eight-year Program, 2202 Class, 2022 Grade, Central South University, Changsha, China; e Department of Geriatrics, The Second Xiangya Hospital of Central South University, Changsha, China; f Postdoctoral Mobile Station of Clinical Medicine, The Second Xiangya Hospital of Central South University, Changsha, China

**Keywords:** Adding salt to foods, Osteoporosis, Genetic susceptibility, UK Biobank

## Abstract

**Objectives:**

To investigate the association between the frequency of adding salt to foods and incident osteoporosis and assess effect modification by genetic susceptibility.

**Design:**

Prospective cohort study.

**Setting:**

UK Biobank, United Kingdom.

**Participants:**

491,428 participants free of osteoporosis at baseline.

**Measurements:**

Frequency of adding salt to foods (excluding salt used in cooking) was assessed by questionnaire and categorized as never/rarely, sometimes, usually, or always. Multivariable Cox models estimated hazard ratios (HRs) and 95% confidence intervals (CIs) for incident osteoporosis and secondary cardiovascular outcomes.

**Results:**

During a median follow-up of 14.7 years, 15,953 osteoporosis cases were documented. Compared with the never/rarely group, fully adjusted HRs were 1.09 (95% CI 1.04–1.15) for usually and 1.32 (95% CI 1.24–1.41) for always (*P* for trend <0.001). The association was stronger in men, White participants, and those with higher BMI. In joint analyses, participants with high genetic risk who always added salt had the highest risk (HR 2.68, 95% CI 2.41–2.98). In the same cohort, always adding salt was also associated with higher cardiovascular mortality (HR 1.18, 95% CI 1.14–1.23) and cardiovascular events (HR 1.21, 95% CI 1.17–1.26).

**Conclusion:**

A higher frequency of adding salt to foods was associated with a higher risk of incident osteoporosis. Together with the cardiovascular associations observed in this cohort, these findings suggest that adding salt to foods may be a modifiable behavior relevant to multiple age-related diseases.

## 1 Introduction

Osteoporosis is a common bone disease characterized by the loss of bone mass and increased bone fragility [1]. With the rapid aging of the global population, it has become a substantial public health burden associated with considerable morbidity and mortality [[2], [3], [4]]. Given suboptimal adherence to antiresorptive therapy and concerns about rare but serious adverse events [[5], [6], [7]], identifying modifiable risk factors for osteoporosis remains an important public health priority.

High sodium intake has been associated with several age-related conditions, including hypertension and cardiovascular disease, chronic kidney disease, metabolic dysfunction, and premature mortality [8,9,22]. Because aging is a major shared risk factor for these conditions, sodium exposure may be relevant to geroscience as a potentially modifiable factor spanning several age-related diseases. Among the available measures of sodium exposure, “adding salt to foods” is a simple behavior-based marker of a person’s long-term preference for salty taste, largely independent of the sodium contained in processed foods, and recent cohort studies have linked this behavior to steatotic liver disease and premature mortality [9,22]. However, its association with bone health has remained unexplored.

Experimental and clinical studies have shown that increased sodium intake can enhance urinary calcium excretion and may promote bone resorption [[10], [11], [12], [13], [14]], whereas findings from epidemiological studies remain inconsistent [[15], [16], [17], [18]]. In the present study, we therefore prospectively investigated the association between the frequency of adding salt to foods and the risk of incident osteoporosis in the UK Biobank, and further examined whether it differed according to genetic susceptibility. We additionally analyzed cardiovascular outcomes in the same cohort.

## 2 Methods

### 2.1 Study population

We used data from the UK Biobank, which recruited over 500,000 adults aged 40–69 years during 2006–2010 [19]. The study was approved by the North West Multicentre Research Ethics Committee (11/NW/0382). From 502,366 individuals, we excluded 1,127 without exposure information, 2,790 with hospital-diagnosed osteoporosis and 7,021 with self-reported osteoporosis at baseline, leaving 491,428 for the primary analysis (Supplementary Fig. S1).

### 2.2 Exposure assessment

The frequency of adding salt to foods (excluding salt used in cooking) was assessed at baseline using a touchscreen questionnaire, with response options of never/rarely, sometimes, usually, always, or prefer not to answer; those who preferred not to answer were treated as missing.

### 2.3 Outcome ascertainment

Incident osteoporosis was identified from linked hospital inpatient records using International Classification of Diseases, 10th Revision (ICD-10) codes M80-M82 [20]. Participants were followed from recruitment until incident osteoporosis, death, or December 19, 2022, whichever occurred first. We additionally examined total, hip, and vertebral fractures, defined from hospital records as detailed in Supplementary Methods. Four secondary cardiovascular outcomes (cardiovascular mortality, myocardial infarction mortality, stroke mortality, and composite cardiovascular events) were ascertained from hospital and death registry records.

### 2.4 Genetic risk score

An osteoporosis genetic risk score (GRS), computed from genome-wide association study effect sizes [21], was obtained from the UK Biobank and stratified into tertiles of genetic susceptibility (low, intermediate, and high), consistent with a previous study [20].

### 2.5 Covariates

Baseline covariates included age, sex, ethnicity, Townsend deprivation index (TDI), smoking status, alcohol intake, body mass index (BMI), and vitamin D and calcium intake; healthy diet score (0–5) and physical activity were defined as previously described [20].

### 2.6 Statistical analysis

Cox proportional hazards regression models estimated HRs and 95% CIs, with the never/rarely group as the reference and the proportional-hazards assumption assessed using Schoenfeld residuals. Four models were constructed with progressive adjustment: Model 1, unadjusted; Model 2, age and sex; Model 3, further adjusted for ethnicity, TDI, healthy diet score, smoking, alcohol, BMI, and physical activity; and Model 4 additionally included vitamin D and calcium intake and was used for all subsequent analyses. Missing data was imputed with mean value for continuous variables and an indicator category for categorical predictors. We conducted subgroup and joint analyses with the GRS, applied the same models to the fracture and cardiovascular outcomes, and performed three sensitivity analyses: excluding cases occurring within the first 2 years, restricting analyses to complete cases, and using multiple imputation. Analyses used SAS 9.4 (SAS Institute, Cary, NC). Two-sided *P* < 0.05 was considered statistically significant.

## 3 Results

### 3.1 Baseline characteristics

Of the 491,428 participants, 272,375 (55.4%) never or rarely added salt to foods, and 137,954 (28.1%), 57,253 (11.6%), and 23,846 (4.9%) did so sometimes, usually, or always, respectively (Supplementary Table S1). A higher frequency of salt addition was associated with higher BMI, less physical activity, lower healthy diet scores, and lower intakes of calcium and vitamin D.

### 3.2 Adding salt to foods and incident osteoporosis

During a median follow-up of 14.7 years, 15,953 osteoporosis cases were documented. We observed a positive graded association between the frequency of adding salt to foods and incident osteoporosis (Table 1). Compared with the never/rarely group, the fully adjusted HRs were 1.09 (95% CI 1.04–1.15) for usually and 1.32 (95% CI 1.24–1.41) for always (*P*-trend <0.001). The association was similar across adjustment models and persisted throughout follow-up (Supplementary Fig. S2). A higher frequency of adding salt to foods was also associated with total and vertebral fractures, but not hip fractures (Supplementary Table S9).

**Table 1:** Association between the frequency of adding salt to foods and incident osteoporosis.

	Adding salt to foods				*P*-trend	*P*-interaction
	Never/Rarely (n = 272,375)	Sometimes (n = 137,954)	Usually (n = 57,253)	Always (n = 23,846)		
Cases/person-years	8,704/3,950,410	4,372/2,002,351	1,915/831,214	962/344,906		
Incidence rate*	2.2 (2.1−2.2)	2.2 (2.1−2.2)	2.3 (2.2−2.4)	2.8 (2.6−3.0)		
Overall						
Model 1	1.00 (Ref)	0.99 (0.96−1.03)	1.05 (1.00−1.10)	1.27 (1.19−1.36)	<0.001	
Model 2	1.00 (Ref)	1.02 (0.98−1.06)	1.11 (1.06−1.17)	1.43 (1.34−1.53)	<0.001	
Model 3	1.00 (Ref)	1.02 (0.98−1.06)	1.09 (1.04−1.15)	1.32 (1.23−1.41)	<0.001	
Model 4	1.00 (Ref)	1.02 (0.98−1.06)	1.09 (1.04−1.15)	1.32 (1.24−1.41)	<0.001	
**By sex**						<0.001
Female, n = 264,495 (53.82%)	1.00 (Ref)	1.01 (0.97−1.05)	1.08 (1.02−1.14)	1.22 (1.13−1.32)	<0.001	
Male, n = 226,933 (46.18%)	1.00 (Ref)	1.06 (0.97−1.16)	1.17 (1.04−1.30)	1.67 (1.46−1.91)	<0.001	
**By ethnicity**						0.02
White, n = 445,186 (90.91%)	1.00 (Ref)	1.03 (0.99−1.07)	1.12 (1.06−1.18)	1.36 (1.26−1.46)	<0.001	
Others, n = 44,517 (9.09%)	1.00 (Ref)	0.95 (0.84−1.08)	0.86 (0.73−1.03)	1.14 (0.93−1.39)	0.88	
**By BMI**						0.04
Normal weight, n = 157,749 (32.43%)	1.00 (Ref)	1.04 (0.98−1.10)	1.07 (0.99−1.17)	1.26 (1.12−1.41)	<0.001	
Overweight, n = 208,339 (42.83%)	1.00 (Ref)	0.96 (0.91−1.02)	1.08 (0.99−1.17)	1.34 (1.20−1.50)	<0.001	
Obesity, n = 120,333 (24.74%)	1.00 (Ref)	1.12 (1.03−1.21)	1.21 (1.09−1.35)	1.37 (1.19−1.58)	<0.001	

* Incidence rate per 1,000 person-years. Model 1: unadjusted; Model 2: age and sex; Model 3: age, sex, ethnicity, TDI, healthy diet score, smoking, alcohol, BMI, and physical activity; Model 4: Model 3 plus vitamin D and calcium intake. Subgroup analyses were adjusted as in Model 4 except for the stratifying variable. Ref, reference. BMI was categorized as normal (18.5–24.9 kg/m^2^), overweight (25.0–29.9 kg/m^2^), or obesity (≥30.0 kg/m^2^). Participants with BMI < 18.5 kg/m^2^ were excluded from the BMI subgroup analysis.

### 3.3 Subgroup and joint analyses

**Fig. 1:**
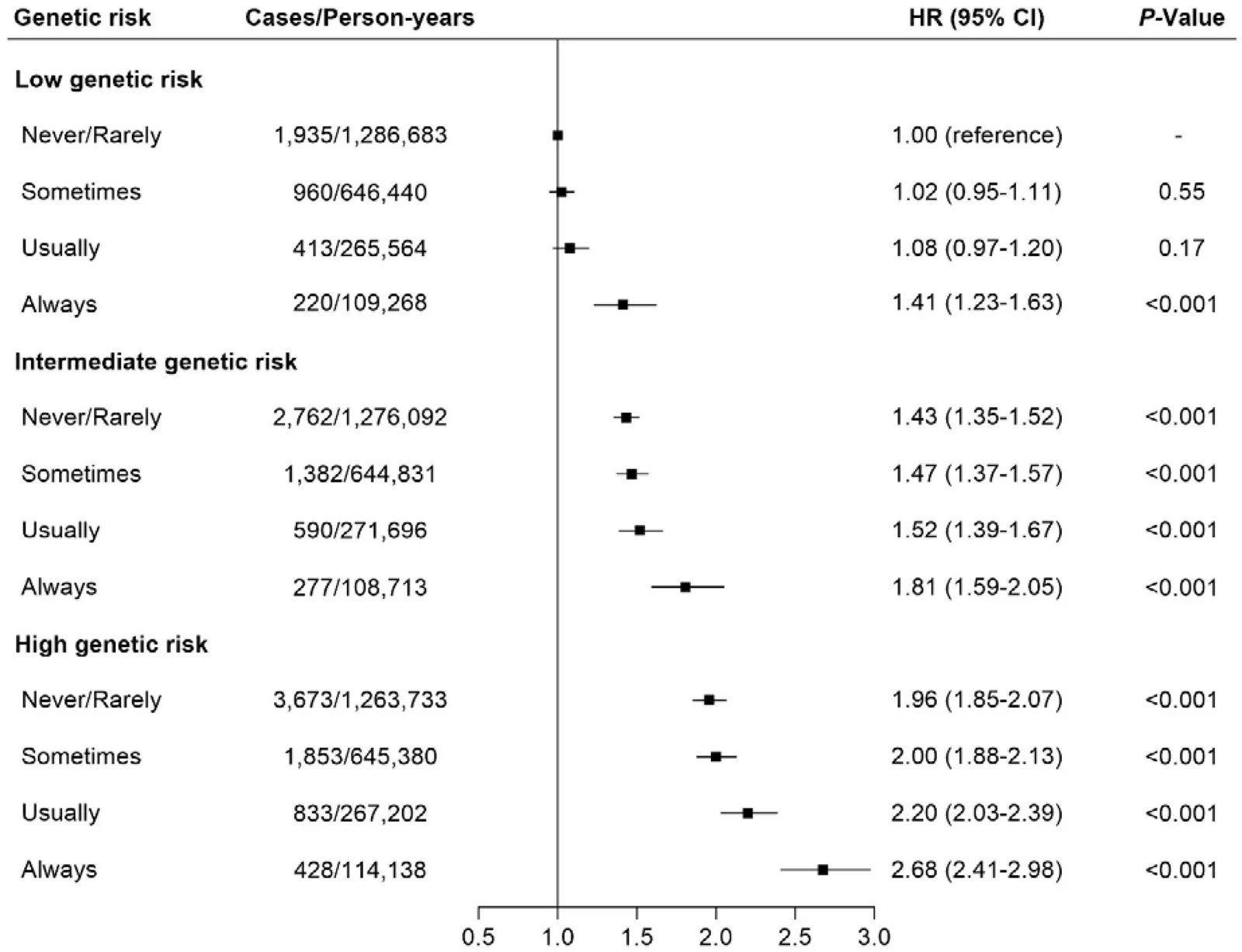
Joint association of the frequency of adding salt to foods and osteoporosis genetic risk score with incident osteoporosis. The model was adjusted for age, sex, ethnicity, TDI, healthy diet score, smoking, alcohol, BMI, physical activity, and vitamin D and calcium intake.

The association was stronger in men (HR 1.67, 95% CI 1.46–1.91 vs 1.22, 95% CI 1.13–1.32 in women; *P*-interaction <0.001), White participants (*P*-interaction = 0.02), and those with higher BMI (*P*-interaction = 0.04) (Table 1; Supplementary Table S2). In joint analyses (Fig. 1), participants with high genetic risk who always added salt had the highest risk (HR 2.68, 95% CI 2.41–2.98) compared with those with low genetic risk who never/rarely added salt. Sensitivity analyses yielded similar results (Supplementary Tables S5-S7).

### 3.4 Adding salt to foods and cardiovascular outcomes

Compared with participants who never or rarely added salt, those who always added salt had higher risks of cardiovascular mortality (HR 1.18, 95% CI 1.14–1.23), myocardial infarction mortality (HR 1.20, 95% CI 1.12–1.28), stroke mortality (HR 1.17, 95% CI 1.08–1.26), and composite cardiovascular events (HR 1.21, 95% CI 1.17–1.26) in the fully adjusted model (all *P*-trend ≤0.007; Supplementary Table S8).

## 4 Discussion

In this large prospective cohort study, a higher frequency of adding salt to foods was associated with a graded increase in the risk of incident osteoporosis, with consistent findings across sensitivity analyses. The association was stronger in men, White participants, and those with higher BMI, while participants with high genetic risk who always added salt had the highest risk. To the best of our knowledge, this is the first prospective study to explore this association. Our findings are consistent with previous evidence linking high sodium intake to poorer bone health. A meta-analysis of nearly 40,000 participants reported a 20% higher risk of osteoporosis with high dietary sodium intake, particularly in older populations [15]. The parallel associations with total and vertebral fractures further support the consistency of the findings across skeletal outcomes, although no association was observed with hip fracture.

### 4.1 Adding salt to foods from a geroscience perspective

Our findings may also be relevant from a geroscience perspective. Adding salt to foods has already been associated with hypertension and cardiovascular disease, chronic kidney disease, metabolic dysfunction-associated steatotic liver disease, and premature mortality [8,9,22], and the present study adds incident osteoporosis to this list. Notably, within the same cohort, we also observed positive graded associations with cardiovascular mortality, myocardial infarction and stroke mortality, and composite cardiovascular events (Supplementary Table S8). These findings indicate that the same modifiable behavior is associated with both skeletal and cardiovascular outcomes within a single cohort. Part of this pleiotropy may arise from shared upstream processes that accompany biological aging, such as activation of the renin-angiotensin-aldosterone system, oxidative stress, endothelial dysfunction, and chronic low-grade inflammation (“inflammaging”), which can affect the vasculature, kidney, liver, and skeleton simultaneously [8,30]. Bone-specific mechanisms, such as sodium-related urinary calcium loss, may additionally contribute to skeletal risk [11,23]. Since adding salt to foods is a modifiable behavior, it may represent a simple behavioral target relevant to healthy aging.

### 4.2 Potential mechanisms

Several biological pathways may explain the observed association with bone. First, higher sodium exposure can reduce calcium reabsorption in the renal tubules, thereby increasing urinary calcium loss and contributing to chronic negative calcium balance and progressive bone loss [23]. Second, a recent animal experiment suggested that excess salt intake may shift bone remodeling toward resorption by altering ion-channel expression in bone tissue [24]. Moreover, frequent salt addition may also reflect a broader unhealthy dietary pattern, such as a preference for highly processed, energy-dense foods, which could further contribute to skeletal risk.

### 4.3 Subgroup and gene-environment findings

The stronger associations in specific subgroups suggest that the skeletal consequences of adding salt to foods may be amplified in vulnerable subgroups. In men, osteoporosis is often under-recognized and insufficiently prevented in clinical practice, which may allow harmful exposures to accumulate for longer before detection [25]. In White participants, the stronger association may partly reflect a less favorable calcium economy, with greater urinary calcium loss under salt loading [[26], [27], [28]]. In participants with higher BMI, chronic low-grade inflammation and insulin resistance may compromise bone remodeling [29,30]. Participants with high genetic risk and frequent salt addition had the highest risk, highlighting a subgroup with a particularly unfavorable combined risk profile. Because salt-adding behavior is modifiable, it may represent a pragmatic target among individuals with elevated genetic susceptibility.

### 4.4 Strengths and limitations

Our study had several strengths, including its prospective design, large sample, extended follow-up, and robustness across sensitivity analyses. Several limitations also warrant consideration. (1) The exposure was assessed by a single self-reported question at baseline and does not quantify total sodium intake. (2) Residual confounding cannot be fully excluded given the observational design. (3) Because exposure and covariates were measured at baseline, changes over time could not be fully captured. (4) The non-significant interaction test suggests that the joint association with genetic risk should not be overinterpreted as evidence of a gene-environment interaction. (5) The predominantly White European cohort may limit generalizability. (6) Reliance on inpatient records may have missed osteoporosis cases or fractures managed without hospitalization.

## 5 Conclusions

Our findings indicate that a higher frequency of adding salt to foods is associated with a higher risk of incident osteoporosis, with stronger associations in men, White participants, and those with higher BMI. Participants with high genetic risk who always added salt had the highest risk. Together with the cardiovascular associations observed in this cohort, these findings suggest that adding salt to foods may be a modifiable behavior relevant to multiple age-related diseases.

## CRediT authorship contribution statement

Xukun Luo: Conceptualization, Formal analysis, Investigation, Writing – original draft, Writing – review & editing. Jiayin Li: Writing – review & editing. Hongxuan Chen: Writing – review & editing. Juan Duan: Writing – review & editing. Tang Liu: Writing – review & editing. Jian Zhou: Conceptualization, Formal analysis, Investigation, Writing – original draft, Writing – review & editing, Supervision.

## Ethics approval and consent to participate

The UK Biobank study was approved by the North West Multicentre Research Ethics Committee (11/NW/0382).

## Patient and public involvement

Participants were not involved in the design, conduct, or reporting of the study.

## Declaration of Generative AI and AI-assisted technologies in the writing process

No generative AI was used in the preparation of this manuscript.

## Funding

This work was supported by the 10.13039/501100012166National Key Research and Development Program of China (Grant No. 2023YFC2507601), the Central South University Research Programme of Advanced Interdisciplinary Studies (Grant No. 2023QYJC026), Hunan Provincial Natural Science Foundation General Program Youth C Project (Grant No. 2026JJ60651), Hunan Provincial Natural Science Foundation General Program (Grant No. 2024JJ30440), Postdoctoral Fellowship Program (Grade C) of China Postdoctoral Science Foundation (Grant No. GZC20251563), China Postdoctoral Science Foundation General Program (Grant No. 2025M782313) and The Second Xiangya Hospital of Central South University Postdoctoral Research Start-up Fund. The study funders/sponsors had no role in the design and conduct of the study; collection, management, analysis, and interpretation of the data; preparation, review, or approval of the manuscript; and decision to submit the manuscript for publication.

## Data availability

This research was conducted using the UK Biobank Resource (https://www.ukbiobank.ac.uk) under Application Number 80610.

## Declaration of competing interest

The authors declare that they have no known competing financial interests or personal relationships that could have appeared to influence the work reported in this paper.
