# Supplementary material for: Adding salt to foods, genetic susceptibility, and risk of incident osteoporosis
Source: J Nutr Health Aging. 2026 Sep 5;30(11):100973. doi: 10.1016/j.jnha.2026.100973 (PMC13571995; doi:10.1016/j.jnha.2026.100973)
Supplement: Supplementary file 1 [file mmc1.docx]

**Supplementary Materials**

**Supplementary Methods.** Ascertainment of bone fractures.

**Supplementary Fig.S1.** Flow chart.

**Supplementary Fig. S2.** The cumulative hazard and cumulative incidence of osteoporosis by frequency of adding salt to foods.

**Supplementary Table S1.** Baseline characteristics of included participants.

**Supplementary Table S2.** Association between the frequency of adding salt to foods and incident risk of osteoporosis stratified by potential risk factors.

**Supplementary Table S3.** Assessment of healthy diet score in the UK Biobank.

**Supplementary Table S4.** The numbers and percentages of participants with missing covariates.

**Supplementary Table S5.** Association between the frequency of adding salt to foods and incident risk of osteoporosis after excluding participants who were diagnosed with osteoporosis during the first two years of follow-up.

**Supplementary Table S6.** Association between the frequency of adding salt to foods and incident risk of osteoporosis after excluding participants with missing covariate data.

**Supplementary Table S7.** Association between the frequency of adding salt to foods and incident risk of osteoporosis after inputting all missing covariates data using multiple imputation.

**Supplementary Table S8.** Association between the frequency of adding salt to foods and cardiovascular outcomes.

**Supplementary Table S9.** Association between the frequency of adding salt to foods and bone fractures.

**Supplementary Methods**

**Ascertainment of bone fractures**

Incident fractures were ascertained from linked hospital inpatient records based on International Classification of Diseases, 10th Revision (ICD-10) codes. Total fractures included hip, vertebral, and other eligible fractures. We excluded fractures of the skull, face, hands, and feet, as well as malignancy-related fractures, atypical femoral fractures, periprosthetic fractures, and healed fractures. Participants with a history of fracture at baseline, identified from either hospital records or self-report, were excluded from the fracture analyses.

**Supplementary Fig. S1.** Flow chart.

**Supplementary Fig. S2.** The cumulative hazard curve (left) and cumulative incidence curve (right) of osteoporosis by frequency of adding salt to foods.

**Supplementary Table S1**. Baseline characteristics of included participants.

| **Characteristics** | **Adding Salt to Foods** | | | |
| --- | --- | --- | --- | --- |
|  | **Never/Rarely (n=272,375)** | **Sometimes (n=137,954)** | **Usually (n=57,253)** | **Always (n=23,846)** |
| Age, years, mean (SD) | 56.4 (8.1) | 56.3 (8.0) | 56.9 (8.0) | 55.8 (8.3) |
| Female, n (%) | 151,072 (55.5) | 73,681 (53.4) | 27,621 (48.2) | 12,115 (50.8) |
| Body mass index, mean (SD) | 27.2 (4.7) | 27.7 (4.8) | 27.9 (4.8) | 28.1 (5.1) |
| Ethnic background, n (%) | | |  |  |
| White | 250,338 (92.2) | 124,130 (90.3) | 50,681 (88.8) | 20,037 (84.4) |
| Others | 21,076 (7.8) | 13,379 (9.7) | 6,366 (11.2) | 3,696 (15.6) |
| Townsend deprivation index, mean (SD) | -1.5 (3.0) | -1.2 (3.1) | -1.1 (3.2) | -0.2 (3.5) |
| Smoking status, n (%) | | | |  |
| Never | 160,883 (59.2) | 71,965 (52.4) | 25,708 (45.1) | 9,562 (40.3) |
| Previous | 88,708 (32.7) | 49,768 (36.2) | 22,536 (39.5) | 8,515 (35.9) |
| Current | 21,875 (8.1) | 15,648 (11.4) | 8,770 (15.4) | 5,626 (23.7) |
| Alcohol intake frequency, n (%) | | | |  |
| Never | 86,228 (31.7) | 40,177 (29.1) | 15,725 (27.5) | 8,240 (34.6) |
| Previous | 71,701 (26.3) | 36,085 (26.2) | 13,793 (24.1) | 5,413 (22.8) |
| Current | 114,273 (42.0) | 61,551 (44.7) | 27,662 (48.4) | 10,140 (42.6) |
| Physical activity, min/week, n (%) | | |  |  |
| <150 | 90,450 (40.3) | 45,118 (40.8) | 19,727 (42.8) | 8,313 (46.0) |
| ≥150 | 133,952 (59.7) | 65,406 (59.2) | 26,391 (57.2) | 9,752 (54.0) |
| Healthy diet score, n (%) | | |  |  |
| <3 | 76,950 (29.3) | 46,392 (35.3) | 22,809 (42.0) | 10,858 (49.5) |
| ≥3 | 185,905 (70.7) | 85,010 (64.7) | 31,553 (58.0) | 11,096 (50.5) |
| Regular calcium intake, n (%) | | |  |  |
| Yes | 17,502 (6.5) | 8,244 (6.0) | 3,194 (5.6) | 1,276 (5.4) |
| No | 252,209 (93.5) | 128,265 (94.0) | 53,441 (94.4) | 22,211 (94.6) |
| Regular vitamin D intake, n (%) | | |  |  |
| Yes | 7,809 (2.9) | 3,772 (2.8) | 1,431 (2.5) | 529 (2.3) |
| No | 261,385 (97.1) | 132,403 (97.2) | 55,037 (97.5) | 22,881 (97.7) |

**Supplementary Table S2.** Association between the frequency of adding salt to foods and incident osteoporosis stratified by potential risk factors.

Subgroup analyses were adjusted as in Model 4 except for the stratifying variable.

| **Subgroup** | **Adding Salt to Foods** | | | | ***P***-**trend** | ***P-*interaction** |
| --- | --- | --- | --- | --- | --- | --- |
|  | **Never/Rarely (n=272,375)** | **Sometimes (n=137,954)** | **Usually (n=57,253)** | **Always (n=23,846)** |  |  |
| Age |  |  |  |  |  | 0.51 |
| <60 | 1.00 (1.00-1.00) | 1.01 (0.95-1.08) | 1.11 (1.02-1.21) | 1.41 (1.26-1.57) | <0.001 |  |
| ≥60 | 1.00 (1.00-1.00) | 1.02 (0.98-1.07) | 1.09 (1.02-1.16) | 1.27 (1.17-1.39) | <0.001 |  |
| Sex |  |  |  |  |  | <0.001 |
| Female | 1.00 (1.00-1.00) | 1.01 (0.97-1.05) | 1.08 (1.02-1.14) | 1.22 (1.13-1.32) | <0.001 |  |
| Male | 1.00 (1.00-1.00) | 1.06 (0.97-1.16) | 1.17 (1.04-1.30) | 1.67 (1.46-1.91) | <0.001 |  |
| Ethnic |  |  |  |  |  | 0.02 |
| White | 1.00 (1.00-1.00) | 1.03 (0.99-1.07) | 1.12 (1.06-1.18) | 1.36 (1.26-1.46) | <0.001 |  |
| Others | 1.00 (1.00-1.00) | 0.95 (0.84-1.08) | 0.86 (0.73-1.03) | 1.14 (0.93-1.39) | 0.88 |  |
| Townsend deprivation index | | |  |  |  | 0.18 |
| <median | 1.00 (1.00-1.00) | 1.05 (1.00-1.10) | 1.12 (1.04-1.20) | 1.28 (1.15-1.42) | <0.001 |  |
| ≥median | 1.00 (1.00-1.00) | 0.98 (0.93-1.04) | 1.07 (0.99-1.15) | 1.33 (1.21-1.45) | <0.001 |  |
| Body Mass Index | |  |  |  |  | 0.04 |
| Normal | 1.00 (1.00-1.00) | 1.04 (0.98-1.10) | 1.07 (0.99-1.17) | 1.26 (1.12-1.41) | <0.001 |  |
| Overweight | 1.00 (1.00-1.00) | 0.96 (0.91-1.02) | 1.08 (0.99-1.17) | 1.34 (1.20-1.50) | <0.001 |  |
| Obesity | 1.00 (1.00-1.00) | 1.12 (1.03-1.21) | 1.21 (1.09-1.35) | 1.37 (1.19-1.58) | <0.001 |  |
| Alcohol intake | |  |  |  |  | 0.20 |
| Never | 1.00 (1.00-1.00) | 1.00 (0.94-1.06) | 1.08 (1.00-1.17) | 1.23 (1.10-1.37) | <0.001 |  |
| Previous | 1.00 (1.00-1.00) | 1.01 (0.94-1.09) | 1.04 (0.93-1.16) | 1.33 (1.15-1.55) | 0.01 |  |
| Current | 1.00 (1.00-1.00) | 1.05 (0.99-1.12) | 1.14 (1.06-1.23) | 1.43 (1.28-1.59) | <0.001 |  |
| Smoking status | |  |  |  |  | 0.18 |
| Never | 1.00 (1.00-1.00) | 1.03 (0.98-1.08) | 1.11 (1.03-1.20) | 1.19 (1.06-1.33) | <0.001 |  |
| Previous | 1.00 (1.00-1.00) | 1.01 (0.95-1.07) | 1.10 (1.02-1.19) | 1.39 (1.24-1.55) | <0.001 |  |
| Current | 1.00 (1.00-1.00) | 1.02 (0.91-1.13) | 1.05 (0.92-1.19) | 1.37 (1.19-1.58) | <0.001 |  |
| Healthy diet score | |  |  |  |  | 0.14 |
| <3 | 1.00 (1.00-1.00) | 1.00 (0.93-1.07) | 1.13 (1.03-1.24) | 1.39 (1.25-1.56) | <0.001 |  |
| ≥3 | 1.00 (1.00-1.00) | 1.02 (0.98-1.07) | 1.09 (1.02-1.16) | 1.27 (1.16-1.40) | <0.001 |  |
| Physical activity level (min/week) | |  |  |  |  | 0.33 |
| <150 | 1.00 (1.00-1.00) | 1.07 (1.00-1.14) | 1.12 (1.03-1.22) | 1.38 (1.23-1.54) | <0.001 |  |
| ≥150 | 1.00 (1.00-1.00) | 0.99 (0.94-1.05) | 1.11 (1.03-1.20) | 1.31 (1.17-1.47) | <0.001 |  |
| Regular vitamin D intake |  |  |  |  |  | 0.07 |
| No | 1.00 (1.00-1.00) | 1.02 (0.98-1.06) | 1.10 (1.04-1.16) | 1.35 (1.26-1.45) | <0.001 |  |
| Yes | 1.00 (1.00-1.00) | 1.08 (0.94-1.24) | 1.05 (0.85-1.29) | 0.84 (0.58-1.22) | 0.93 |  |
| Regular calcium intake |  |  |  |  |  | 0.82 |
| No | 1.00 (1.00-1.00) | 1.02 (0.98-1.06) | 1.11 (1.05-1.17) | 1.37 (1.27-1.47) | <0.001 |  |
| Yes | 1.00 (1.00-1.00) | 1.03 (0.94-1.13) | 1.06 (0.93-1.20) | 1.09 (0.90-1.32) | 0.24 |  |

**Supplementary Table S3.** Assessment of healthy diet score in the UK Biobank.

| **Diet** | **UK Biobank field ID** | **Description** | **Healthy diet score** |
| --- | --- | --- | --- |
| Vegetable | 1289 | Cooked vegetable intake | 1 for ≥4 tablespoons/day  0 for <4 tablespoons/day |
|  | 1299 | Salad/raw vegetable intake |  |
| Fruit | 1309 | Fresh fruit intake | 1 for ≥3 pieces/day  0 for <3 pieces/day |
|  | 1319 | Dried fruit intake |  |
| Fish | 1329 | Oily fish intake | 1 for ≥2 times/week  0 for <2 times/week |
|  | 1339 | Non-oily fish intake |  |
| Processed meat | 1349 | Processed meat intake | 1 for ≤2 times/week  0 for >2 times/week |
| Unprocessed red meat | 1369 | Beef intake | 1 for ≤2 times/week  0 for >2 times/week |
|  | 1379 | Lamb/mutton intake |  |
|  | 1389 | Pork intake |  |

**Supplementary Table S4.** The numbers and percentages of participants with missing covariates.

| **Variable** | **N** | **%** |
| --- | --- | --- |
| Physical activity | 92,319 | 18.78 |
| Healthy diet score | 20,855 | 4.24 |
| Regular vitamin D intake | 6,181 | 1.26 |
| Regular calcium intake | 5,086 | 1.03 |
| Body mass index | 2,596 | 0.53 |
| Smoking status | 1,864 | 0.38 |
| Ethnic background | 1,725 | 0.35 |
| Townsend deprivation index | 614 | 0.12 |
| Alcohol intake frequency | 440 | 0.09 |

**Supplementary Table S5.** Association between the frequency of adding salt to foods and incident osteoporosis after excluding participants who were diagnosed with osteoporosis during the first two years of follow-up.

Cox proportional hazards models 1–4 were constructed as follows: Model 1 without covariate adjustment; Model 2 adjusted for age and sex; Model 3 adjusted for age, sex, ethnicity, TDI, healthy diet score, smoking status, alcohol intake, BMI, and physical activity; and Model 4 additionally adjusted for vitamin D and calcium intake based on Model 3.

|  | **Adding Salt to Foods** | | | | ***P*-trend** |
| --- | --- | --- | --- | --- | --- |
|  | **Never/Rarely (n=271,855)** | **Sometimes (n=137,676)** | **Usually (n=57,141)** | **Always (n=23,787)** |  |
| Model 1 | 1.00 (1.00-1.00) | 0.99 (0.96-1.03) | 1.05 (1.00-1.11) | 1.27 (1.18-1.36) | <0.001 |
| Model 2 | 1.00 (1.00-1.00) | 1.02 (0.98-1.06) | 1.11 (1.06-1.17) | 1.43 (1.33-1.53) | <0.001 |
| Model 3 | 1.00 (1.00-1.00) | 1.02 (0.98-1.06) | 1.10 (1.05-1.16) | 1.32 (1.23-1.42) | <0.001 |
| Model 4 | 1.00 (1.00-1.00) | 1.02 (0.98-1.06) | 1.10 (1.05-1.16) | 1.33 (1.24-1.42) | <0.001 |

**Supplementary Table S6.** Association between the frequency of adding salt to foods and incident osteoporosis after excluding participants with missing covariate data.

Cox proportional hazards models 1–4 were constructed as follows: Model 1 without covariate adjustment; Model 2 adjusted for age and sex; Model 3 adjusted for age, sex, ethnicity, TDI, healthy diet score, smoking status, alcohol intake, BMI, and physical activity; and Model 4 additionally adjusted for vitamin D and calcium intake based on Model 3.

|  | **Adding Salt to Foods** | | | | ***P*-trend** |
| --- | --- | --- | --- | --- | --- |
|  | **Never/Rarely (n=217,034)** | **Sometimes (n=106,237)** | **Usually (n=44,090)** | **Always (n=16,900)** |  |
| Model 1 | 1.00 (1.00-1.00) | 0.99 (0.96-1.03) | 1.05 (1.00-1.10) | 1.27 (1.19-1.36) | <0.001 |
| Model 2 | 1.00 (1.00-1.00) | 1.02 (0.98-1.06) | 1.11 (1.06-1.17) | 1.43 (1.34-1.53) | <0.001 |
| Model 3 | 1.00 (1.00-1.00) | 1.02 (0.98-1.07) | 1.11 (1.05-1.18) | 1.33 (1.22-1.44) | <0.001 |
| Model 4 | 1.00 (1.00-1.00) | 1.02 (0.98-1.07) | 1.11 (1.05-1.18) | 1.33 (1.23-1.45) | <0.001 |

**Supplementary Table S7.** Association between the frequency of adding salt to foods and incident osteoporosis after inputting all missing covariates data using multiple imputation.

Cox proportional hazards models 1–4 were constructed as follows: Model 1 without covariate adjustment; Model 2 adjusted for age and sex; Model 3 adjusted for age, sex, ethnicity, TDI, healthy diet score, smoking status, alcohol intake, BMI, and physical activity; and Model 4 additionally adjusted for vitamin D and calcium intake based on Model 3.

|  | **Adding Salt to Foods** | | | | ***P*-trend** |
| --- | --- | --- | --- | --- | --- |
|  | **Never/Rarely (n=272,375)** | **Sometimes (n=137,954)** | **Usually (n=57,253)** | **Always (n=23,846)** |  |
| Model 1 | 1.00 (1.00-1.00) | 0.99 (0.96-1.03) | 1.05 (1.00-1.10) | 1.27 (1.19-1.36) | <0.001 |
| Model 2 | 1.00 (1.00-1.00) | 1.02 (0.98-1.06) | 1.11 (1.06-1.17) | 1.43 (1.34-1.53) | <0.001 |
| Model 3 | 1.00 (1.00-1.00) | 1.02 (0.98-1.07) | 1.11 (1.05-1.18) | 1.33 (1.22-1.44) | <0.001 |
| Model 4 | 1.00 (1.00-1.00) | 1.02 (0.98-1.07) | 1.11 (1.05-1.18) | 1.33 (1.23-1.45) | <0.001 |

**Supplementary Table S8.** Association between the frequency of adding salt to foods and cardiovascular outcomes.

Cox proportional hazards models 1–4 were constructed as follows: Model 1 without covariate adjustment; Model 2 adjusted for age and sex; Model 3 adjusted for age, sex, ethnicity, TDI, healthy diet score, smoking status, alcohol intake, BMI, and physical activity; and Model 4 additionally adjusted for vitamin D and calcium intake based on Model 3.

|  | **Adding Salt to Foods** | | | | ***P*-trend** |
| --- | --- | --- | --- | --- | --- |
|  | **Never/Rarely (n=272,375)** | **Sometimes (n=137,954)** | **Usually (n=57,253)** | **Always (n=23,846)** |  |
| Cardiovascular mortality | 1.00 (1.00-1.00) | 1.03 (1.01-1.05) | 1.03 (1.00-1.06) | 1.18 (1.14-1.23) | <0.001 |
| Myocardial infarction mortality | 1.00 (1.00-1.00) | 1.02 (0.98-1.06) | 1.05 (1.01-1.11) | 1.20 (1.12-1.28) | <0.001 |
| Stroke mortality | 1.00 (1.00-1.00) | 1.03 (0.98-1.07) | 1.01 (0.95-1.07) | 1.17 (1.08-1.26) | 0.007 |
| Cardiovascular events | 1.00 (1.00-1.00) | 1.03 (1.01-1.05) | 1.04 (1.01-1.07) | 1.21 (1.17-1.26) | <0.001 |

**Supplementary Table S9.** Association between the frequency of adding salt to foods and bone fractures.

Cox proportional hazards models 1–4 were constructed as follows: Model 1 without covariate adjustment; Model 2 adjusted for age and sex; Model 3 adjusted for age, sex, ethnicity, TDI, healthy diet score, smoking status, alcohol intake, BMI, and physical activity; and Model 4 additionally adjusted for vitamin D and calcium intake based on Model 3.

|  | **Adding Salt to Foods** | | | | ***P*-trend** |
| --- | --- | --- | --- | --- | --- |
|  | **Never/Rarely (n=246,471**) | **Sometimes (n=124,191)** | **Usually (n=51,127)** | **Always (n=21,084)** |  |
| Total fractures |  |  |  |  |  |
| Model 1 | 1.00 (1.00-1.00) | 1.03 (1.00-1.06) | 1.09 (1.05-1.13) | 1.21 (1.14-1.28) | <0.001 |
| Model 2 | 1.00 (1.00-1.00) | 1.04 (1.01-1.07) | 1.10 (1.06-1.15) | 1.27 (1.20-1.35) | <0.001 |
| Model 3 | 1.00 (1.00-1.00) | 1.02 (0.99-1.05) | 1.06 (1.02-1.11) | 1.17 (1.11-1.24) | <0.001 |
| Model 4 | 1.00 (1.00-1.00) | 1.02 (1.00-1.05) | 1.06 (1.02-1.11) | 1.17 (1.11-1.24) | <0.001 |
| Hip fractures |  |  |  |  |  |
| Model 1 | 1.00 (1.00-1.00) | 0.96 (0.89-1.02) | 1.03 (0.94-1.13) | 1.14 (1.00-1.30) | 0.23 |
| Model 2 | 1.00 (1.00-1.00) | 0.97 (0.91-1.04) | 1.02 (0.93-1.12) | 1.23 (1.08-1.40) | 0.06 |
| Model 3 | 1.00 (1.00-1.00) | 0.96 (0.90-1.03) | 0.98 (0.89-1.08) | 1.09 (0.95-1.24) | 0.82 |
| Model 4 | 1.00 (1.00-1.00) | 0.96 (0.90-1.03) | 0.98 (0.89-1.08) | 1.09 (0.95-1.25) | 0.81 |
| Vertebral fractures |  |  |  |  |  |
| Model 1 | 1.00 (1.00-1.00) | 1.08 (0.99-1.19) | 1.24 (1.09-1.40) | 1.61 (1.37-1.88) | <0.001 |
| Model 2 | 1.00 (1.00-1.00) | 1.09 (1.00-1.20) | 1.20 (1.06-1.35) | 1.68 (1.43-1.97) | <0.001 |
| Model 3 | 1.00 (1.00-1.00) | 1.04 (0.95-1.14) | 1.10 (0.97-1.24) | 1.39 (1.18-1.64) | <0.001 |
| Model 4 | 1.00 (1.00-1.00) | 1.04 (0.95-1.14) | 1.10 (0.97-1.24) | 1.40 (1.19-1.64) | <0.001 |
